# Testing a new cognitive behavioural treatment for obesity: A randomized controlled trial with three-year follow-up

**DOI:** 10.1016/j.brat.2010.03.008

**Published:** 2010-08

**Authors:** Zafra Cooper, Helen A. Doll, Deborah M. Hawker, Susan Byrne, Gillie Bonner, Elizabeth Eeley, Marianne E. O’Connor, Christopher G. Fairburn

**Affiliations:** Oxford University, Department of Psychiatry, Warneford Hospital, Oxford OX3 7JX, United Kingdom

**Keywords:** Obesity, Treatment, Cognitive behaviour therapy, Behaviour therapy, Self-help, Randomized controlled trial

## Abstract

It is remarkably difficult for people with obesity to maintain a new lower weight following weight loss. The aim of the present study was to examine the immediate and longer-term effects of a new cognitive behavioural treatment that was explicitly designed to minimise this post-treatment weight regain. One hundred and fifty female participants with obesity were randomized to the new treatment, behaviour therapy (the leading alternative psychological treatment) or guided self-help (a minimal intervention). Both of the main treatments resulted in an average weight loss of about ten percent of initial weight whereas weight loss was more modest with guided self-help. The participants were subsequently followed-up for three years post-treatment. The great majority regained almost all the weight that they had lost with the new treatment being no better than the behavioural treatment in preventing weight regain. These findings lend further support to the notion that obesity is resistant to psychological methods of treatment, if anything other than a short-term perspective is taken. It is suggested that it is ethically questionable to claim that psychological treatments for obesity “work” in the absence of data on their longer-term effects.

## Introduction

Most people who seek treatment for obesity are able to lose weight, but few are able to sustain the changes in behaviour required to prevent subsequent weight regain. As a result the majority regain much of the weight that they have lost (Cussler et al., 2008; Jones, Wilson, & Wadden, 2007; Turk et al., 2009). While subsequent therapeutic support of various forms appears to delay this weight regain, the effects are modest and do not persist in most cases (Svetkey et al., 2008; Wing et al., 2006).

Advances in the field of eating disorders might be of relevance to this problem of weight regain as it has been established that certain psychological treatments result in many of these patients making lasting changes to the way that they eat. Of particular relevance is the research on bulimia nervosa, an eating disorder characterized by recurrent episodes of uncontrolled overeating (binge eating) and one that tends to run a chronic course. This research has identified a clear leading treatment, a specific form of cognitive behaviour therapy (CBT-BN; Fairburn, 2008; Fairburn, Marcus, and Wilson, 1993) that addresses not only these patients’ overeating but also the processes that are hypothesized to maintain it. CBT-BN results in a substantial decrease in the frequency of binge eating and related behaviour with up to half the patients ceasing to binge eat altogether, and the treatment effect persists in the majority of cases (Wilson and Fairburn, 2007). Similar findings are emerging from research on binge eating disorder, another eating disorder characterized by repeated binge eating and one that often coexists with obesity (Wilson and Fairburn, 2007).

Recently a new cognitive behavioural approach to the treatment of obesity has been developed that is similar in style and strategy to CBT-BN. It not only targets these patients’ overeating and low level of activity but it also focuses on processes hypothesized to hinder successful weight maintenance (Cooper and Fairburn, 2001, 2002). The aim of the present study was to evaluate this new form of CBT. To this end, it was compared with the leading alternative psychological treatment for obesity, behaviour therapy (BT). In addition, the short-term effects of both treatments were compared with those of a minimal intervention, guided self-help (GSH). It was predicted that BT and CBT would be superior to GSH at producing weight loss in the short-term and that CBT would be superior to BT at preventing subsequent weight regain. To address this latter aim, patients were followed-up for three years post-treatment. A subsidiary aim of the study was to explore the relationship between binge eating, weight loss and weight regain.

## Methods

### Trial design

One hundred and fifty women with obesity were randomly assigned to CBT, BT or GSH. Those in the CBT and BT conditions received treatment over 44 weeks, whereas GSH took 24 weeks. Each participant was followed-up for three years from week 44 (i.e., the end of the CBT and BT conditions). No further treatment was provided after the end of treatment (week 24 for those in GSH; week 44 for those in BT and CBT). The sample size was determined *a priori* to provide sufficient power (80%) to detect a 30% difference at two-sided *p* < 0.05 between CBT and BT in the amount of weight regained at follow-up.

The study protocol was approved by the Local Institutional Review Board. Before being enrolled in the study, each potential participant provided written informed consent to the senior clinical investigator (ZC or CGF) who had previously explained the nature and purpose of the study and had provided an approved information sheet.

### Participants

Potential participants were either referred by their family physician or they contacted us directly in response to advertisements placed in physicians’ clinics and local hospitals. They were eligible to take part if they met the following criteria: (1) female, (2) aged between 20 and 60 years, (3) body mass index (weight in kg/height in m^2^; BMI) between 30.0 and 39.9, (4) available for treatment for 44 weeks, and (5) willing to participate in the study. The exclusion criteria were: (1) weight loss of 10% or more within the previous six months, (2) major medical or psychiatric illness (including Type I or Type II diabetes), (3) current psychiatric or psychological treatment, and (4) disorders or treatments known to affect eating, weight or metabolic rate, and disorders in which calorie or fat restriction are contraindicated. Those receiving treatment for hypertension or hypercholesterolemia were eligible to take part provided their condition had been stable on medication over the previous three months. Participants who reported binge eating were eligible to take part.

**Fig. 1:**
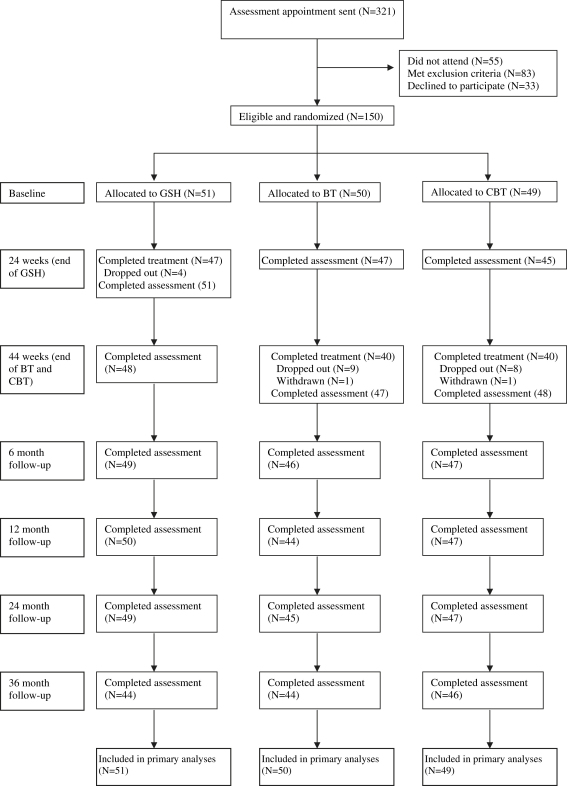
CONSORT diagram.

Three hundred and twenty-one potential participants were offered an assessment interview. Fifty-five did not attend and a further 83 were ineligible. Thirty-three elected not to take part. Fig. 1 shows the CONSORT diagram.

### Treatments

#### Cognitive behaviour therapy (CBT)

The new form of CBT was designed to address certain psychological processes that had been hypothesized to interfere with successful weight maintenance (Cooper and Fairburn, 2001, 2002). Briefly, it was proposed that weight regain is a result of failure to engage in effective weight maintenance behaviour and that this largely stems from a progressive within-treatment decrease in individuals’ belief that they can control their weight to a worthwhile extent. This in turn stems from the almost invariable decline in the rate of weight loss experienced after four to six months of attempting to lose weight, together with the growing realization that they will not obtain the benefits that they hoped would result from losing weight (e.g., markedly improved appearance). As a result they abandon their attempts to lose further weight and instead return to their prior eating and exercising habits with the consequence that they regain the weight that they have lost. Thus the goal of the new treatment was not only to produce weight loss but also to help people accept and value more modest changes in weight and appearance. The treatment was also designed to encourage the acquisition and practice of weight maintenance skills as these differ from those required to lose weight.

As evaluated in the present study, CBT comprised 24, 50-min, one-to-one sessions over the 44 week period of treatment with the sessions being weekly for the first seven weeks and every two weeks thereafter. The weight loss phase lasted for the first 24–30 weeks (during which participants were helped to restrict their energy intake to about 1500 kcal daily) with the remainder of treatment being devoted to the establishment of weight maintenance skills. Full details of the treatment have been published elsewhere (Cooper, Fairburn, and Hawker, 2003).

#### Behaviour therapy (BT)

BT was based on the Pittsburgh Behavioural Weight Control Manual (Wing, 1995) and the Weight Maintenance Guide of Wadden and colleagues (Wadden and Foster 1989). It was designed to represent the optimal behavioural treatment available at the time, adapted for use on an individual basis. The style of the treatment was that of modern behaviour therapy with the treatment being applied flexibly so as to match the individual’s needs and progress. It involved the same number of sessions as CBT and the same pattern of appointments. Well-established behavioural methods were used to help participants change their eating habits and activity level, the aim being that they restrict their energy intake to 1200 kcal daily. Between weeks 24 and 30, and again at week 36, the subject of weight maintenance was raised and participants were given the choice of either continuing to pursue further weight loss for the remainder of treatment or deciding to maintain their new lower weight.

#### Guided self-help (GSH)

This treatment was based on the LEARN Programme for Weight Control (seventh edition) (Brownell, 1997), a widely used weight loss program. The LEARN programme is designed to produce permanent change in five areas of life: lifestyle, exercise, attitudes, relationships and nutrition. Participants are asked to restrict their energy intake to 1200 kcal daily, make healthy food choices, and gradually increase their level of activity. GSH involved participants following the LEARN programme with a limited amount of guidance and support from a therapist. This mode of treatment delivery was based on our experience using guided self-help in the treatment of eating disorders (Carter & Fairburn, 1998). GSH lasted 24 weeks and involved two initial face-to-face sessions with a therapist followed by up to 15 20-min telephone sessions.

#### Therapists and supervision

There were three therapists and each delivered all three treatments after a six-month period of training. All three treatments were fully manualised. Two of the therapists were clinical psychologists and one was a dietician. Throughout the study there was weekly group supervision led by the two senior clinicians. All sessions were recorded and regularly audited to ensure that the treatments were well implemented.

#### Assessment

Basic demographic and historical information was collected at baseline by the assessing clinician. Thereafter the assessments were conducted by independent assessors who were blind to the participants’ treatment conditions. The in-treatment assessments took place at the beginning of treatment, and at 24 and 44 weeks (end of GSH, and CBT and BT respectively). The follow-up assessments took place 6, 12, 24 and 36 months after the 44-week assessment. Weight was measured using a beam balance scale and height was measured using an eye level wall-mounted stadiometer.

Participants were interviewed using an adaptation of the Eating Disorder Examination (Fairburn, Cooper, & O’Connor, 2008) that included additional items designed to assess acceptance of shape and the practice of weight maintenance behaviour post-treatment, both targets of CBT, as well as the presence and frequency of binge eating. General psychiatric features were measured using the Brief Symptom Inventory (BSI; Derogatis & Spencer, 1982) a short form of the Symptom Checklist (SCL-90; Derogatis, 1977). Quality of life was measured using the Medical Outcomes Study 36 item Short Form Health Survey (SF-36) Questionnaire (McHorney, Ware, & Reczek, 1993; Ware & Sherbourne, 1992). The SF-36 is a multi-dimensional self-report questionnaire measuring general quality of life by assessing 8 dimensions including positive and negative aspects of health. Two summary component scores of mental (MCS) and physical (PCS) well-being can also be calculated. It has been shown to have good psychometric properties in a British sample (Jenkinson, Layte, & Lawrence, 1997).

### Randomization

Participants were allocated to the three treatments by HAD (who had no involvement in participant recruitment) using a stratified computer-generated randomization scheme with random permuted blocks of varying size within two strata. Participants were assigned to the two strata on the basis of their binge eating frequency with those reporting 12 or more episodes over the previous 12 weeks being classed as belonging to a binge eating subgroup. The allocation sequence was concealed in numbered sealed opaque envelopes. At the point of randomization, the next envelope in the sequence was opened by one of the two senior clinicians.

### Statistical analysis

Data were first examined to check for outliers and to determine the data distribution. Following missing value imputation (see below), change scores were calculated for weight, BMI, binge eating frequency, acceptance of shape and weight from both baseline and end of treatment to each follow-up point. Data are presented as *N* (%) for categorical data and mean (SD) for continuous data. Analyses of variance with post hoc tests (or non-parametric Kruskal–Wallis, as appropriate) and *t*-tests (or non-parametric Mann–Whitney tests) were used to compare continuous scores (both absolute and change scores) at each assessment point; chi-squared tests were used to compare categorical variables. Analyses of covariance were used to adjust continuous scores for baseline values. To compare BT and CBT with respect to weight change following treatment, a series of multi-level models (see Singer and Willett, 2003) were fitted to the data. These models allow for correlation between repeated measurements and for both fixed and random effects. Fixed effects were fitted for treatment condition, baseline weight, time, and the interaction between treatment condition and time. Results were similar whether or not a random intercept was included for subject.

All analyses were performed on an intent-to-treat basis. The missing data were imputed using a method for repeated measures with dropouts (Little and Rubin, 2002). Each missing data point was taken as the within-treatment group median of the distribution of possible scores calculated for each participant with valid data at that point. Each possible score was the valid data point for each participant weighted by the difference between the mean of all other data points (i.e., overall times) for that participant and the participant with missing data. Results were, however, similar if simpler methods of imputation were used, such as substituting the mean value of the two surrounding data points. Statistical significance was taken at the 5% level (*p* < 0.05) throughout, with 95% confidence intervals (CIs) used to express the uncertainty in the estimates.

## Results

### Sample and attrition

The demographic and clinical characteristics of the sample are shown in Table 1. The three treatment groups were similar with respect to age, marital status, weight history and family history of obesity. However, they differed with regard to baseline weight and BMI, with those assigned to CBT having a lower mean(SD) weight (92.3 kg (8.8 kg)) and consequently BMI at the start of treatment than those who were allocated to either BT (95.2 kg (11.2 kg)) or GSH (95.9 kg (9.2 kg)). As there is evidence of a positive association between pre-treatment weight and absolute weight change after treatment (Latner, Wilson, Jackson, & Stunkard, 2009; Teixeira, Going, Sardinha, & Lohman, 2005), subsequent analyses were adjusted for baseline weight.

Twenty-one participants (14%) did not complete treatment (4 GSH (8%), 9 BT (18%), 8 CBT (16%); *χ*^2^ = 2.49, df = 2, *p* = 0.29) and two were withdrawn on medical grounds, one each from CBT and BT. Compliance with the assessment protocol was high. Nine hundred and twenty-one of the 1050 assessments (87.7%) were conducted face-to-face with objective weight data being obtained. There were no differences between the treatment groups in their compliance with the assessment protocol (K-W *χ*^2^ = 0.58, df = 2, *p* = 0.75).

### Weight change during treatment

Mean weight, adjusted weight (weight adjusted for baseline weight) and percentage weight change for the three treatment conditions at each time point is shown in Table 2. Also shown are the number of participants who achieved 5% and 10% weight loss at each assessment point and the number of participants who achieved a 5% and 10% weight loss after treatment and maintained this degree of weight loss at each successive follow-up point. At 24 weeks (end of GSH), the mean percentage weight losses were 6.7%, 11.3%, and 10.0%, respectively, in the GSH, BT, and CBT conditions (ANOVA *F* = 5.90, df = 2,147, *p* = 0.003). Those who received BT lost significantly more weight than those in GSH (mean difference 4.60%; post hoc Tukey test *p* = 0.003) but there was no statistically significant difference between BT and CBT (mean difference 1.38%, *p* = 0.58). At 44 weeks (end of BT and CBT) mean percentage weight losses were 5.4%, 12.7% and 8.9% respectively (ANOVA *F* = 9.39, df = 2,147, *p* < 0.001). Again, participants who received BT had lost significantly more weight than those who had received GSH (mean difference 7.29%, *p* < 0.001), with the difference between BT and CBT not being statistically significant (mean difference 3.80%, *p* = 0.07). With regard to the period between 24 and 44 weeks there was a significant increase in weight among the participants in both the GSH and CBT conditions (mean weight gain in GSH 1.18 kg, *t* = 3.10, df = 50, *p* = 0.003, 95% CI 0.41 to 1.95 kg; mean weight gain in CBT 0.97 kg, *t* = 2.94, df = 48, *p* = 0.005, 95% CI 0.31 to 1.62 kg) whereas there was a small statistically significant decrease in weight among the BT participants (mean weight loss 1.31 kg, *t* = 2.33, df = 49, *p* = 0.024, 95% CI = 2.45 to 0.18 kg), with the overall difference between groups being statistically significant at the 0.1% level (ANOVA *F* = 10.05, df = 2,147, *p* < 0.001).

At 44 weeks the participants had lost on average 9.0% of their initial weight with over half (60.7%) having lost 5% or more, and over a third (36.0%) 10% or more. The percentage of participants who had lost 5% or more of their initial weight at 44 weeks was greater among those who received BT and CBT than among those who received GSH (76%, 71% and 35% respectively; *χ*^2^ = 21.1, df = 2, *p* < 0.001), as was the percentage who had lost 10% or more (54% in BT, 37% in CBT, 18% in GSH; *χ*^2^ = 14.5, df = 2, *p* = 0.001).

## Maintenance of weight change following BT and CBT

**Fig. 2:**
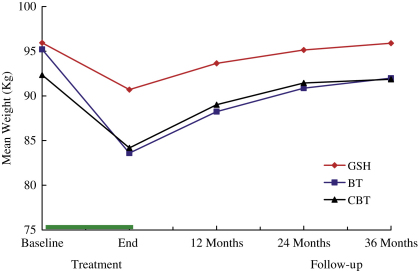
Mean weight (kg) during treatment and three-year follow-up in participants receiving either cognitive behaviour therapy (CBT), behaviour therapy (BT) or guided self-help (GSH).

Most of the BT and CBT participants regained weight following the end of treatment (see Table 2 and Fig. 2). Almost all (*N* = 98/99, 99%) had a weight higher than their end of treatment weight at some point during the three years following treatment (data not shown). At 1-year follow-up, those who had lost weight at the end of treatment had regained, overall, almost half the weight that they had lost (median regain of weight lost, 43.5% in BT and 58.0% in CBT, M–W *z* = 0.07, *p* = 0.94), and at 3-year follow-up they had regained almost all the weight lost (89.8% regain in BT; 88.6% regain in CBT; M–W *z* = 0.63, *p* = 0.53). The overall proportion of participants who were 5% or more below their initial weight at three-year follow-up was greater among those who received BT than those who received CBT, but this difference was not statistically significant (38.0% *n* = 19/50 vs 24.5%, *n* = 12/49 *χ*^2^ = 1.52, df = 1, *p* = 0.22). A similar non-significant difference was also observed in terms of the proportion of participants who were 10% or more below their initial weight (22.0% *n* = 11/50 vs. 8.2% *n* = 4/49; *χ*^2^ = 2.69, df = 1, *p* = 0.11). There were also no significant differences between those who received BT and CBT in terms of the proportion of participants who successfully maintained either 5% (24.0% *n* = 12/50 vs 16.3% *n* = 8/49 *χ*^2^ = 0.49, df = 1, *p* = 0.48) or 10% (12.0% *n* = 6/50 vs 2.0% *n* = 1/49, *χ*^2^ = 2.37, df = 1, *p* = 0.12) weight loss across each follow-up point.

To compare CBT and BT with respect to weight change during follow-up a series of multi-level models were fitted to the data. Initial weight and follow-up time (*p* < 0.001 in both cases) were the strongest predictors of follow-up weight, with follow-up weight on average being 0.95 (SE 0.06) of baseline weight and with weight increasing from baseline on average by 1.66 (SE 0.08) kg at every assessment. There was a significant (*p* = 0.04) adjusted mean difference in follow-up weight between participants in the CBT and BT conditions (2.79 kg in favour of BT; 95% CI 0.13–5.45). Although participants in the BT condition gained weight slightly less quickly than those in the CBT condition (by 0.26 (SE 0.20) kg on average at each assessment), the interaction between treatment condition and time was not statistically significant (*p* = 0.19) indicating that the rate of weight regain did not differ significantly between the two treatments.

### Targeted psychological and behavioural change

The new form of CBT was designed to enhance participants’ acceptance of their shape and encourage their implementation of weight maintenance behaviour (an index of this being the regular monitoring of body weight). In both conditions, at the end of treatment the greater the weight loss, and in particular the greater the percentage weight loss, the higher the acceptance of shape (overall *r*_s_ = 0.37 and 0.50, respectively, both *p* < 0.001). After adjusting for baseline acceptance of shape and percentage weight change during treatment, those who received CBT were significantly more accepting of their shape than those who received BT (adjusted means mid-treatment (potential range 0 to 6, where 0 indicates greater acceptance): 3.26 vs 2.41, *F* = 12.4, df = 1,95, *p* = 0.001; adjusted means at end treatment 2.32 vs 2.85, *F* = 4.62, df = 1,95, *p* = 0.034). There was no significant difference between the two conditions with respect to their frequency of weighing (*p* = 0.16).

During follow-up the beneficial effect of CBT relative to BT with respect to acceptance of shape persisted (repeated measures ANOVA, adjusting for baseline acceptance of shape, effect of group *F* = 3.24, df = 1,96, *p* = 0.075), with there being a progressive increase in acceptance of shape in CBT but not BT (interaction term *F* = 4.32, df = 1,96, *p* = 0.040). While there was a significant relationship across and within both groups between acceptance of shape at end of treatment and weight during follow-up, with the greater the acceptance the lower the weight (*F* = 22.2, df = 1,96, *p* < 0.001), this did not result in an advantage for CBT in terms of maintenance of the weight lost (*F* = 0.032, df = 1,96, *p* = 0.86) since the association between follow-up weight and actual post-treatment weight was stronger: after adjusting for post-treatment weight there was no association between acceptance of shape and follow-up weight (repeated measures ANOVA *F* = 0.12, df = 1,95, *p* = 0.74).

### Binge eating, weight loss and weight regain

Thirty-six participants (24%) reported episodes of binge eating at baseline and 14 (9.3%) were categorized as belonging to the binge eating subgroup (with 12 or more episodes of binge eating over 12 weeks). Six participants (4%) met the DSM-IV research criteria for binge eating disorder (APA, 1994). The three treatment conditions were similar with respect to the proportions of participants who reported any binge eating, binge eating at least weekly and those meeting diagnostic criteria for binge eating disorder. The number of participants who reported binge eating decreased during treatment to 24 (16.0%) at 44 weeks. Six (42.9%) of the 14 participants in the binge eating subgroup ceased binge eating and the binge eating subgroup lost on average 11.6% of their baseline weight at 44 weeks compared with 9.1% among the remainder (M–W *p* = 0.60). Nine (7.9%) of the 114 participants who reported no episodes of binge eating at baseline reported some binge eating at 44 weeks.

At 3-year follow-up 25 (16.7%) participants reported any binge eating compared with the 36 (24%) at the start of treatment. Of these, seven belonged to the original binge eating subgroup (with 12 or more episodes over 3 months). Thus half (*N* = 7) of those in the original binge eating group (*N* = 14) reported no binge eating at 3-year follow-up. The participants in the original binge eating subgroup had an average weight loss of 4.18% of their baseline weight compared with 0.96% among the remainder (M–W *p* = 0.22).

### General psychiatric features and quality of life

Treatment had a beneficial effect on patients’ psychiatric symptoms and their quality of life. There was a statistically significant improvement from baseline to the end of treatment in BSI scores across all treatment groups (mean(SD) improvement = 0.30(0.43), *t* = 8.50, df = 148, *p* < 0.001). Adjusting for pre-treatment BSI scores and weight change, those receiving CBT achieved slightly lower scores on the BSI than those receiving BT but the difference was not statistically significant (mean(SE) scores 0.48(.05) vs 0.51(.05), *F* = 0.31, *p* = 0.58). Across all three groups there were statistically significant improvements in the eight SF-36 domains and the two summary scores (PCS and MCS) from baseline to the end of treatment (mean(SD) improvement PCS 3.96(9.23), *t* = 5.20, *p* < 0.001; MCS 5.43(9.21), *t* = 7.15, *p* < 0.001), although generally there were statistically significant deteriorations in scores during follow-up (mean(SD) deterioration PCS 3.20(10.2) *t* = 3.13, *p* = 0.002; MCS 2.40(7.53), *p* = 0.002). Adjusting for pre-treatment score and change in weight during treatment and follow-up, there were no significant differences in the extent of these changes between patients receiving BT and CBT with adjusted PCS and MCS scores being comparable both at the end of treatment (mean(SE) PCS 49.4(1.18) vs 49.1(1.19), *F* = 0.04, *p* = 0.85; MCS 48.9(1.05) vs 49.1(1.06), *F* = 0.02, *p* = 0.90) and at follow-up, although there was some evidence of a slight advantage in favour of CBT at this point (mean(SE) PCS 44.9(1.18) vs 47.1(1.20), *F* = 1.63, *p* = 0.20; MCS 46.8(0.97) vs 46.3(0.99), *F* = 0.10, *p* = 0.75)

## Comment

The aim of the present study was to evaluate a new form of CBT for obesity by comparing it with the leading alternative psychological treatment, behaviour therapy (BT) and with a minimal intervention, a form of guided self-help (GSH). The immediate and longer-term effects of the treatments were studied. The treatment completion rates were high as was the level of compliance with the assessment protocol even up to three years post-treatment. Thus the study achieved what it set out to do; namely, to provide a rigorous test of the new form of treatment.

The findings are clear. The great majority of the participants lost weight and then regained it and CBT, despite being explicitly designed to prevent post-treatment weight regain, was no better than BT in this regard. This is particularly evident in the low proportion of patients in both treatment conditions who were able to maintain either a 5% or 10% weight loss throughout follow-up. This lack of difference between CBT and BT was not because they were no different in their impact. The pattern of weight loss in CBT differed from that obtained with BT and was as expected given the nature of the treatment (see Methods above). CBT was also successful at achieving change in participants’ acceptance of shape. Thus, despite realising its aims CBT did not result in improved weight maintenance.

A subsidiary aim of the study was to explore the relationship between binge eating, weight loss and weight regain. This proved difficult as less than ten percent of the sample reported binge eating on a regular basis. What is clear is that weight loss treatment did not promote binge eating, either during treatment or afterwards.

The study had certain strengths that increase confidence in its findings. First, as noted above, the three treatments were acceptable to the participants, achieved different effects, and compliance with the assessment protocol was high. Thus the treatment experiment worked and little data imputation was required. Second, the therapists were well trained and closely supervised. Third, the new treatment was devised by a team that has experience developing psychological interventions that have proved effective as treatments for eating disorders (Fairburn, 1981; Fairburn, Jones, Peveler, Hope, & O’Connor, 1993; Fairburn et al., 2009).

Two main conclusions may be drawn from the findings. Neither is new. The first is that among people with obesity it is remarkably difficult to maintain a new lower weight following weight loss. It can be done (Ikeda et al., 2005; Wing & Phelan, 2005) but it is not common. The reasons for this are not known. It is possible that the processes specified by the CBT theory do indeed operate but that our treatment was not sufficiently effective at changing them. Thus it is not possible to determine from this study whether the theory is incorrect or whether CBT was not sufficiently potent. Alternatively or additionally, other processes may be largely responsible for weight regain.

The second conclusion has far-reaching implications. It stems from the finding that sustained behaviour change in people with obesity is remarkably difficult to achieve, unlike the situation with people with eating disorders (e.g., Fairburn et al., 2009). This is a sufficiently robust finding to make it ethically questionable to claim that psychological treatments for obesity “work” in the absence of data on their longer-term outcome. A further implication is that psychosocial research on obesity should perhaps shift away from work on treatment and instead focus on prevention.

## Figures and Tables

**Table 1:** Demographic and clinical characteristics of the full sample and the three treatment groups.

	Total sample (*N* = 150) % or *M* (SD)	GSH (*N* = 51) % or *M* (SD)	BT (*N* = 50) % or *M* (SD)	CBT (*N* = 49) % or *M* (SD)
Age (years)	41.49 (9.07)	41.86 (8.67)	41.38 (9.90)	41.20 (8.77)
Marital status				
Single/never married	12.7%	9.8%	22.0%	6.1%
Married/cohabiting	74.0%	74.5%	64.0%	83.7%
Widowed	0.7%	2.0%	–	–
Separated/divorced not cohabiting	12.7%	13.7%	14.0%	10.2%
Weight history				
Lowest adult weight (kg)	61.99 (8.13)	62.56 (8.72)	61.58 (6.88)	61.81 (8.79)
Highest adult weight (kg)	96.36 (10.59)	98.10 (11.34)	97.50 (10.58)	93.43 (9.30)
Family history of obesity				
0 first degree relatives	51.3%	52.9%	50.0%	51.0%
1 first degree relative	26.3%	19.6%	30.0%	26.5%
≥2 first degree relatives	23.3%	27.5%	20.0%	22.4%
Weight				
Current weight (kg)	94.04 (9.66)	95.67 (9.44)	94.56 (10.74)	91.80 (8.42)
Body mass index (BMI)	34.69 (2.88)	35.41 (2.71)	34.79 (3.06)	33.85 (2.71)
Binge eating (over past 3 months)				
Any binge eating	24.0%	23.5%	22.0%	26.5%
Weekly binge eating (≥12 episodes)	9.3%	9.8%	10.0%	8.2%
Met criteria for binge eating disorder	4.0%	3.9%	4.0%	4.1%

GSH = Guided self-help; BT = Behaviour therapy; CBT = Cognitive behaviour therapy.

**Table 2:** Weight change during treatment and three-year follow-up in participants receiving either cognitive behaviour therapy (CBT), behaviour therapy (BT) or guided self-help (GSH). Statistical significance is shown for differences among all three study groups unless otherwise stated.

	Baseline	Mid-treatment	End	6 months	12 months	24 months	36 months
*Weight (kg)*							
GSH (*N* = 51)	95.94 (9.18)	89.52 (11.58)	90.70 (11.66)	92.97 (11.65)	93.64 (11.04)	95.14 (11.61)	95.90 (10.89)
BT (*N* = 50)	95.20 (11.15)	84.48 (12.64)	83.60 (14.60)	86.46 (14.38)	88.24 (14.34)	90.86 (12.94)	91.99 (13.43)
CBT (*N* = 49)	92.34 (8.81)	83.20(10.39)	84.17 (11.11)	86.76 (11.21)	89.02 (11.48)	91.44 (11.17)	91.86 (10.69)
Total (*N* = 150)	94.52 (9.83)	85.77 (11.83)	86.05 (12.82)	88.77 (12.77)	90.33 (12.52)	92.50 (12.01)	93.28 (11.81)

*Adjusted weight (kg; adjusted for baseline weight)*							
GSH (*N* = 51)	–	88.14 (6.67)	89.33 (8.22)	91.56 (7.93)	92.21 (7.51)	93.70 (6.76)	94.53 (7.10)
BT (*N* = 50)	–	83.81 (6.65)	82.50 (8.20)	85.78 (7.91)	87.55 (7.49)	90.16 (6.74)	91.33 (7.08)
CBT (*N* = 49)	–	85.32 (6.71)	86.27 (8.26)	88.92 (7.97)	91.22 (7.55)	93.64 (6.78)	93.98 (7.13)
Total (*N* = 150)	–	85.75 (6.65)**	86.03 (8.19)***	88.75 (7.91)**	90.32 (7.48)**	92.50 (6.74)*	93.28 (7.07)
Group differences	–	BT < GSH**	BT < GSH***	BT < GSH*	BT < GSH** BT < CBT*	BT < GSH* BT < CBT*	BT < GSH**

*Percentage weight change from baseline*							
GSH (*N* = 51)	–	−6.74 (7.60)	−5.43 (8.34)	−3.07 (8.05)	−2.35 (7.52)	−0.86 (7.23)	0.05 (7.30)
BT (*N* = 50)	–	−11.33 (7.02)	−12.73 (9.89)	−9.25 (9.77)	−7.42 (9.22)	−4.56 (7.72)	−3.38 (8.27)
CBT (*N* = 49)	–	−9.95 (5.97)	−8.93 (6.82)	−6.12 (6.71)	−3.67 (6.74)	−1.02 (6.48)	−0.44 (7.01)
Total sample (*N* = 150)	–	−9.32 (7.13)**	−9.01 (8.92)***	−6.13 (8.61)**	−4.47 (8.13)**	−2.14 (7.32)*	−1.26 (7.65)

*Weight loss* ≥*5%; N (%)*							
GSH (*N* = 51)	–	26 (51.0%)	18 (35.3%)	10 (19.6%)	11 (21.6%)	11 (21.6%)	9 (17.7%)
BT (*N* = 50)	–	39 (78.0%)	38 (76.0%)	30 (60.0%)	26 (52.0%)	20 (40.0%)	19 (38.0%)
CBT (*N* = 49)	–	39 (79.6%)	35 (71.4%)	29 (59.2%)	20 (40.8%)	11 (22.5%)	12 (24.5%)
Total (*N* = 150)	–	104 (69.3%)**	91 (60.7%)***	69 (46.0%)***	57 (38.0%)**	42 (28.0%)	40 (26.7%)

*Weight loss* ≥*10%; N (%)*							
GSH (*N* = 51)	–	11 (21.6%)	9 (17.6%)	8 (15.7%)	5 (9.8%)	6 (11.8%)	4 (7.8%)
BT (*N* = 50)	–	27 (54.0%)	27 (54.0%)	21 (42.0%)	21 (42.0%)	10 (20.0%)	11 (22.0%)
CBT (*N* = 49)	–	24 (49.0%)	18 (36.7%)	14 (28.6%)	10 (20.4%)	6 (12.2%)	4 (8.2%)
Total (*N* = 150)	–	62 (41.3%)**	54 (36.0%)**	43 (28.7%)*	36 (24.0%)**	22 (14.7%)	19 (12.7%)

*Weight loss >5% at the end of treatment maintained over each successive follow-up point; N (%)*							
GSH (*N* = 51)	–	–	18 (35.3%)	10 (19.6%)	7 (13.7%)	4 (7.8%)	4 (7.8%)
BT (*N* = 50)	–	–	38 (76.0%)	29 (58.0%)	25 (50.0%)	15 (30.0%)	12 (24.0%)
CBT (*N* = 49)	–	–	35 (71.4%)	27 (55.1%)	17 (34.7%)	11 (22.4%)	8 (16.3%)
Total	–	–	91 (60.7%)***	66 (44.0%)***	49 (32.7%)***	30 (20.0%)*	24 (16.0%)

*Weight loss >10% at the end of treatment maintained over each successive follow-up point; N (%)*							
GSH (*N* = 51)	–	–	9 (17.6%)	8 (15.7%)	3 (5.9%)	3 (5.9%)	3 (5.9%)
BT (*N* = 50)	–	–	27 (54.0%)	21 (42.0%)	19 (38.0%)	7 (14.0%)	6 (12.0%)
CBT (*N* = 49)	–	–	18 (36.7%)	13 (26.5%)	6 (12.2%)	4 (8.2%)	1 (2.0%)
Total	–	–	54 (36.0%)**	42 (28.0%) *	28 (18.7%)***	14 (9.3%)	10 (6.7%)

Note: *p < .05; **p < .01; ***p < .001.
